# Assessing Pain Control Efficacy of Meloxicam and Ketoprofen When Compounded with Iron Dextran in Nursing Piglets Using A Navigation Chute

**DOI:** 10.3390/ani10071237

**Published:** 2020-07-21

**Authors:** Kristen Reynolds, Ron Johnson, Jennifer Brown, Robert Friendship, Terri L. O’Sullivan

**Affiliations:** 1 Department of Population Medicine, Ontario Veterinary College, University of Guelph, Guelph, ON N1G 2W1, Canada; kreyno06@uoguelph.ca (K.R.); rfriends@uoguelph.ca (R.F.); 2 Department of Biomedical Science, Ontario Veterinary College, University of Guelph, Guelph, ON N1G 2W1, Canada; rjohns03@uoguelph.ca; 3 Prairie Swine Centre, Saskatoon, SK S7H 5N9, Canada; jennifer.brown@usask.ca

**Keywords:** piglet, castration, analgesia, meloxicam, ketoprofen, iron dextran, compounding

## Abstract

**Simple Summary:**

Post-procedural castration pain control in piglets in Canada is managed with non-steroidal anti-inflammatory drugs (NSAIDs) such as meloxicam and ketoprofen. While combining NSAIDs with iron dextran (ID) reduces piglet injections and handling, it is unknown if this will affect NSAID pain control efficacy. This study evaluated the time it takes a pig to navigate an obstacle chute after surgical castration as an objective measure of pain control. The differences in navigation time of pigs given NSAIDs alone or mixed with ID prior to castration were compared. The results indicate that castrated piglets given either NSAIDs alone or mixed with ID navigated the chute as fast as non-castrated piglets, and faster than castrated piglets not given NSAIDs. This provides evidence that when NSAIDs are combined with iron and administered as a single injection, the NSAIDs remain effective at controlling castration pain.

**Abstract:**

The efficacy of analgesics such as meloxicam and ketoprofen to control pain in piglets when mixed with iron dextran (ID) before injection is unknown. The purpose of this study was to compare perceived pain in castrated piglets treated 1 h before castration with either of these drugs alone, or when mixed with ID, by observing the time it takes for piglets to navigate a chute. Piglets were divided into seven treatment groups (*n* = 25 piglets per treatment group) including castration with analgesia (meloxicam or ketoprofen), castration with analgesic plus ID, castration without analgesic or ID, sham handled and given ID, and sham handled alone. Piglets were placed in a short chute and their time to navigate the chute was recorded at four timepoints following castration. Piglets given meloxicam or ketoprofen, with or without ID did not differ from each other in their chute navigation times. Additionally, these piglets did not differ from treatment groups that were not castrated. Piglets castrated without analgesia had significantly longer navigation times. These results indicate that meloxicam or ketoprofen, whether mixed with ID prior to injection or not, provide similar analgesic efficacy.

## 1. Introduction

The surgical castration of piglets in swine production is currently a routine procedure in Canada and the United States. In Canada specifically, the Code of Practice for the Care and Handling of Pigs [1] states that piglets are to be given analgesia for painful procedures such as surgical castration. Previous research has shown castration in nursing piglets to be both acutely and chronically painful [2,3]. Drugs licensed for use in Canada for the control of pain in swine are currently limited to non-steroidal anti-inflammatory drugs (NSAIDs) such as meloxicam, ketoprofen, and flunixin meglumine. Given as a single injection prior to castration, meloxicam [2,4,5,6,7] and ketoprofen [8,9] have been shown to provide post-castration pain relief to piglets. The labelled doses of meloxicam and ketoprofen (0.4 mg/kg and 3 mg/kg, respectively) are for older pigs. However, studies have evaluated and documented the efficacy of these label doses in smaller (nursing) piglets [2,4,5,6,7,8,9].

There has been interest within the Canadian swine industry to explore the efficacy of mixing NSAIDs and other medications with iron dextran, prior to injection [10]. The mixing of iron dextran and NSAIDs together is a form of compounding, and is formally defined as the customization of any prescription medication by a veterinarian or pharmacist [11]. It is important that the reader be familiar with the laws and regulations specific to the legality of the practice of compounding drugs for use in food-producing animals, as it varies by country/region. While the practice of drug mixing in this manner has potential benefits with regard to increasing production efficiency and decreasing animal handling and stress, the efficacy of the compounded formulations has not been investigated. The mixing of drugs can cause alterations of drug pharmacokinetics and bioavailability [12], and this may in turn limit drug efficacy. Recent work has shown that when meloxicam is compounded with iron dextran prior to administration in piglets before castration, those piglets have lower levels of serum cortisol compared to piglets not receiving analgesia prior to castration [13]. This suggests that pigs receiving the compounded medications experience reduced pain following castration.

Chute navigation time has been utilized as a pain assessment tool to objectively compare piglets castrated with or without analgesia and has been used to investigate meloxicam [14] and sucrose [15] as analgesics. The time required for a piglet to navigate a chute is recorded, and is subject to less misclassification bias than other subjective scoring outcomes. We were interested in applying this assessment tool to our research question.

The objective of this study was to evaluate differences in pain control, as measured by chute navigation time, in piglets administered NSAIDS alone and castrated versus piglets administered NSAIDs compounded with iron dextran and castrated. Specifically, the objectives were to compare chute navigation times of piglets given meloxicam or ketoprofen as a single injection, to the same NSAIDs administered when compounded (mixed together) with iron dextran (ID). The working null hypothesis was that no differences in navigation times (NT) of piglets given NSAIDs alone vs. piglets given NSAIDs compounded with iron dextran would be found.

## 2. Materials and Methods

### 2.1. Animals

This study was conducted at the University of Guelph Swine Research facility (Arkell, ON, Canada). The study was reviewed and approved by the University of Guelph Animal Care Committee (AUP#3744) following the guidelines outlined by the Canadian Council of Animal Care. A total of 175 crossbred male Yorkshire × Landrace × Duroc piglets were enrolled in the study and assigned to one of seven treatment groups resulting in 25 piglets per treatment. Sample size considerations were calculated based on observed differences in chute navigation time from work done by Bilsborrow et al. [14]. Litters at the study facility were born in a 4-week, batch-farrowing schedule. Piglets were selected as they were born and according to the availability of male piglets. Enrolment was conducted with the goal of seven male piglets per litter to have one male piglet per treatment. When seven male piglets per litter were not born naturally, males from other litters, born within 24 h of a target litter, were cross-fostered onto a litter that required additional male piglets. Where possible, an additional eighth male was enrolled in each litter as a potential replacement piglet, should one of the original seven males be censored from the study. Reasons for censoring piglets included injury, smaller than average size, or death. Piglets were assigned a number from 1–7, and this number was marked on their backs using a marker pen (Sharpie^TM^, Newell Office Brands, Atlanta, GA, USA) and marks were reapplied as needed to ensure visibility. Piglet overall health, body condition, and castration site (if applicable) was monitored and recorded daily by the research team. Any health concerns observed were treated according to standard operating procedures of the facility and piglets were censored from study as needed. Additionally, barn staff conducted daily routine health observations.

### 2.2. Chute Training and Back Testing

**Figure 1:**
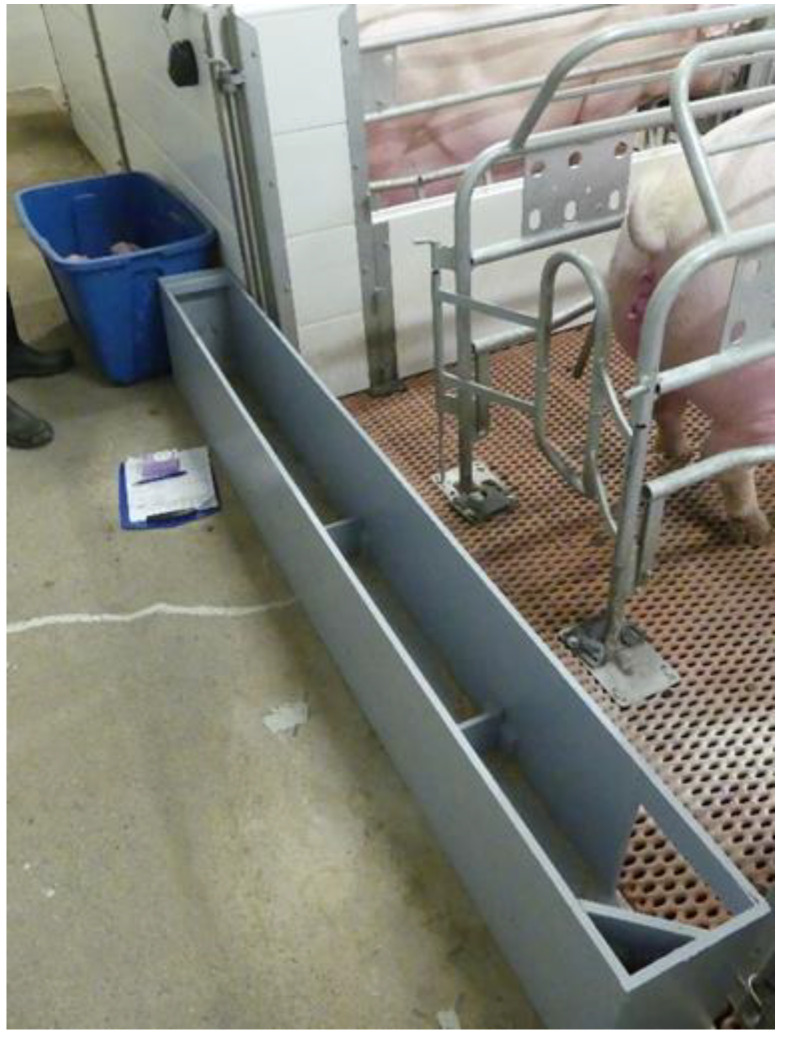
A picture of the navigation chute placed at the back end of the farrowing crate. The blue container at top left is a holding container for piglets prior to placement in the chute. Piglets are placed to start chute navigation in the section closest to this container. Two hurdles visible along the length of the chute, which piglets step over in order to exit the chute. At bottom right is the opening where piglets exit back into the farrowing crate.

The day prior to treatment administration, piglets were trained to negotiate a straight navigation chute with two hurdles spaced along the length of the chute. The chute was placed across the back end of the farrowing crate behind the sow. The chute was portable and could be transferred between crates as needed. The wooden navigation chute (painted for ease of cleaning) was designed according to specifications described by Bilsborrow et al. [14] (width 0.18 m, height 0.33 m, and 0.10 m hurdles) with the minor modification of an additional 0.30 m added lengthwise to result in a total length of 2.07 m (Figure 1). This additional length added to the chute acted as a “starting area” for each piglet, with a line demarcating this area from the rest of the chute.

Piglets were exposed to the navigation chute on four occasions over the course of 30 h to train the piglets to return to their crate and reunite with the dam and littermates. A back-test was also applied to each piglet immediately preceding chute training, and the number of escape attempts made by the piglets was quantified and given a score (0–2, with 0 indicating no escape attempts made) as previously described by Hessing et al. [16]. The back-test score is a potential indicator of an individual piglet’s coping style with regard to stressful situations, and the score was evaluated as a potential covariate in modeling chute navigation time. Observers performing the back-test were trained and practiced prior to applying this test on trial piglets. Results were compared for the back-test observers over the training period and resulted in 100% agreement on multiple practice litters.

### 2.3. Treatment Groups and Treatment Allocation Concealment

Treatment group allocation was conducted by manually drawing a randomized list of treatment groups A–G from an envelope, which were then assigned to piglets. A separate researcher conducted the individual randomizations (www.random.org) for the piglets’ treatment randomization, and the master list was placed in a sealed envelope. Another researcher who was blind to the treatment allocations gave piglets their randomized allocation on treatment day.

The seven treatments administered to piglets were: (1) meloxicam 0.4 mg/kg intramuscular (IM) (Metacam^®^ 20 mg/mL, Boehringer Ingelheim (Canada) Ltd, Burlington, ON, Canada) and castrated (M); (2) ketoprofen 3 mg/kg IM (Anafen^®^ 100 mg/mL, MERIAL Canada Inc, Baie d’Urfé, QC, Canada) and castrated (K); (3) meloxicam 0.4 mg/kg compounded with iron dextran IM (Dexafer-200^®^ 200 mg/mL, Vetoquinol N.A. Inc., Lavaltrie, QC, Canada) and castrated (M + ID); (4) ketoprofen 3 mg/kg compounded with iron dextran IM and castrated (K + ID); (5) iron dextran IM and castrated (C + ID); (6) iron dextran IM and not castrated/sham-handled (ID-C); and (7) no injection and not castrated/sham-handled (SH). Piglets that were sham-handled were restrained for the same amount of time as other piglets, and handled as if they were castrated, though no castration was performed. The doses of meloxicam and ketoprofen were based on the label doses for meloxicam and ketoprofen. These doses have been documented to be effective at reducing post-surgical pain in nursing piglets in similar studies evaluating castration pain [2,4,5,6,7,8,9].

### 2.4. Treatment Preparation and Administration

On the day of treatment and castration, researchers involved in treatment administration (“injection team”) prepared new bottles of the two compounded formulations used (M + ID and K + ID). A new bottle was made each day litters were to be treated and any mixture left over after treatment was discarded. Injectable formulations were pipetted into sterile glass vials. The M + ID formulation combined 0.68 mL of meloxicam (Metacam^®^ 20 mg/mL, Boehringer Ingelheim (Canada) Ltd., Burlington, ON, Canada) with 9.32 mL iron dextran (Dexafer-200^®^ 200 mg/mL, Vetoquinol N.A. Inc., Lavaltrie, QC, Canada) to achieve 1.36 mg meloxicam/mL of compounded formulation. This resulted in a 1.0 mL injection of compounded formulation per 3.4 kg pig, which was the expected maximum weight of piglets at the time of castration. This ensured that a maximum of 1.0 mL of compounded formulation in a tuberculin syringe and needle would be administered for dosing accuracy. The K + ID formulation combined 1.02 mL of ketoprofen (Anafen^®^ 100 mg/mL, MERIAL Canada Inc, Baie d’Urfé, QC, Canada) with 8.98 mL of iron dextran (to result in 1.0 mL per 3.4 kg pig as previously noted). Both compounded formulations were agitated by hand after combining to ensure adequate mixing. From these formulations, pigs received either 0.4 mg/kg meloxicam, or 3 mg/kg ketoprofen, if they were allocated to one of these treatment groups (M + ID or K + ID, respectively). This ensured that the NSAID component of the compounded formulation was being dosed at the same rate as the NSAID given alone (i.e., M and K groups).

Piglets received their allocated treatment at 3–5 days of age, 1 h prior to castration (time = −1 h), organized and timed by multiple calibrated clocks to ensure consistent intervals between the injection team, the barn staff doing castrations (“castration team”), and the researchers performing outcome observations (“observation team”). The injection team gathered piglets minutes before treatment; each piglet’s treatment day weight was recorded, and their injectable formulation (if applicable) was drawn up accordingly. Injections were given intramuscularly in the right side of the neck. Piglets were returned to their farrowing crate following injection. The injection team was timed and organized to perform treatments by litter in rooms where piglets were not being observed for chute navigation time, in order to not startle or interfere with nearby litters of piglets performing chute navigation data collection.

### 2.5. Piglet Castration

Piglets were surgically castrated with a single horizontal incision with a scalpel, following facility standard operating protocols by trained barn staff. Time of castration was coordinated with the same calibrated clocks and pre-determined schedules by litter (time = 0). The castration team removed all study subject piglets from their farrowing crate 5 min prior to castration, and they were taken to a separate nearby room where the castration procedure would not interfere with the observation team and piglet chute navigation. Piglets were either castrated or sham-handled according to a list, given to the castration team by the injection team, generated by the treatment allocations that were determined earlier that morning. Piglets were castrated in order of their number (1–7). Piglets were returned to their farrowing crate immediately after castration. No additional processing procedures (i.e., tail docking, ear notching, teeth clipping, or additional injections) were performed.

### 2.6. Chute Navigation

The observation team placed the navigation chute at the back end of the farrowing crate and collected study piglets into a temporary holding container prior to the scheduled chute observation time (Figure 1). An additional littermate that was not on trial but from the same litter was placed in the same container, so that the last piglet exposed to the chute was not isolated before being placed into the chute. Navigation runs were done at seven timepoints; once prior to castration (time = −0.25 h), and six additional timepoints after castration (time = 0.25 h, 0.5 h, 1.0 h, 4.0 h, 24.0 h, 30.0 h). At each timepoint, piglets were placed individually in the starting area of the chute, in order of piglet number (1–7). The chute navigation time started when a piglet’s first forefoot left the starting area and ended when one of the piglet’s forefeet touched the flooring of the farrowing crate outside of the chute. The navigation time was recorded to the nearest second by an observer with a handheld stopwatch. Once one piglet reached the farrowing crate (finish) and the navigation time was recorded, the next piglet was placed in the chute start area within 30 s. The time to conduct the chute navigation ranged from 2–10 min per litter. After the final chute navigation run, piglets were considered off-trial, except for piglets whose hemoglobin levels were evaluated as part of a separate study.

### 2.7. Cortisol Sampling

Serum samples were collected from all piglets to assess their cortisol levels at 1 h post-castration. Cortisol, as a primary stress hormone, is a common physiological measure observed in studies of pain. We chose to include this outcome in our study to assist with the interpretation of our chute navigation results as well as for a comparison with previous literature. Following the 1.0 h chute navigation timepoint, blood was collected from the orbital sinus of each piglet as previously described by Huhn et al. [17] using an 18-gauge hypodermic needle (Monoject^TM^, Covidien^TM^, Dublin, Ireland), into sterile tubes (BD Vacutainer^®^ Serum Blood Collection Tubes—4 mL, Beckton, Dickinson and Company, Franklin Lakes, NJ, USA). A subset of piglets also had blood collected for hemoglobin analysis at the same time from the same route (as well as three weeks post-castration) as part of a separate study (Reynolds et al., 2020 unpublished). Serum cortisol in piglets has been shown to peak at 30 min post-castration [18]. Due to the design of our navigation timepoints, a 30-min post-castration blood sample was not feasible and given that blood results have been shown to still be elevated at the 1.0 h mark [18], the 1.0 h timepoint was selected. Blood was allowed to clot and kept refrigerated until centrifugation. Samples were centrifuged at 1400 rpm for 20 min, at a temperature of 4 °C (IEC Centra^®^ CL3R, Thermo Electron Corporation, Waltham, MA, USA). Extracted sera were placed in labeled cryovials and stored at −20 °C until the time of analysis of cortisol levels (chemiluminescence, Animal Health Laboratory, University of Guelph, Guelph, Canada).

### 2.8. Statistical Analysis 

Modeling of chute navigation time was evaluated two different ways: as overall mean chute navigation time and by individual timepoint. Overall mean chute navigation time by treatment group (model 1) examined all observations at all time points collectively by using mixed effects linear regression with a restricted maximum likelihood estimation approach and an autoregressive correlation structure to account for the time between repeated measurements (StataIC 14, Statacorp LP, College Station, TX, USA). Univariable associations of treatment and other potential covariates and confounders with overall navigation time were initially investigated. Variables significant at the *p* < 0.10 level were considered for further modeling. Significant univariable associations were added to the model in a forward stepwise fashion as fixed effects or as random intercepts to account for repeated measures. Observation time was evaluated as a random slope as well as for quadratic effect and these were not significant. For covariates with multiple categories, likelihood ratio tests were performed to assess the significance of the overall variable in the model. These steps were performed until an optimal value for Akaike’s information criterion and Bayesian information criterion were achieved. Postestimation diagnostics were performed. Standardized residuals indicated some minor deviations with normality and homoscedasticity, however, transformations of the data (i.e., log transformation) did not improve fit and hence the data were interpreted untransformed. Best linear unbiased predictors were evaluated for the pig level of clustering used in the model, and while there was some mild fanning of higher fitted values, log transformation did not improve fit. Modeling of mean chute navigation time at individual timepoints was conducted as a separate model from overall navigation time (model 2). Individual timepoint models for the four observation times, thus each account for 1/6 of the overall observations. This model was created separately, as the fixed effects of timepoint, for example, did not differ among observations, and thus was not required to be controlled for in the model. In addition, different covariates were important when timepoints were analyzed on their own as opposed to evaluating them together for overall effect of treatment (i.e., effect of treatment over the entire observation period). Individual timepoint modeling was completed using simple linear regression, controlling for covariates and interactions as described above. 

Modeling of serum cortisol for treatment group (model 3) was completed using a linear mixed model, accounting for clustering at the litter level, and controlling for the covariates of treatment day weight (per 100 g difference in weight) and effect of individual castrator. Similarly, univariable associations of treatment as well as other potential covariates/confounders were initially investigated, and variables significant at the *p* < 0.10 level were included in further modeling. Stepwise addition and subtraction of significant univariable associations was performed until an optimal value for Akaike’s information criterion and the Bayesian information criterion were achieved. Postestimation of standardized residuals was unremarkable.

## 3. Results

### 3.1. Descriptive Statistics

Descriptive statistics by treatment for the 175 enrolled piglets are presented in Table 1.

Mean birth weight and mean treatment day weights between treatment group means were not different nor was age at time of treatment (*p* > 0.05). There were some minor differences in average sow parity between treatment groups, but this was not significant in the final model (model 1). The M treatment group had a lower percentage of cross-fostering than other treatment groups, but whether a pig was cross-fostered or not was not associated with chute navigation time in the final model (model 1, model 2).

When evaluating observations done by batch, the number of litters enrolled varied from 3–6 litters. This occurred due to differing available male pigs per batch. One piglet in the second batch was excluded from the final three chute runs due to health concerns.

### 3.2. Chute Navigation Time (Model 1, Model 2)

**Figure 2:**
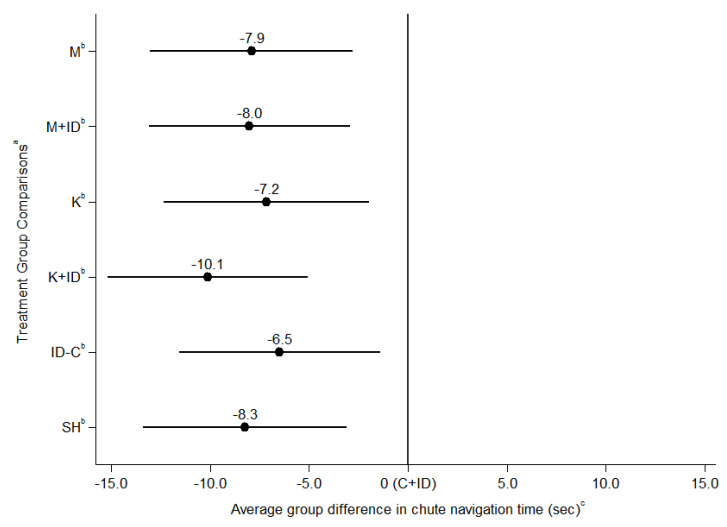
Comparison of overall navigation time (seconds) between treatment groups using mixed effects linear regression, controlling for fixed effects of navigation timepoint, baseline navigation time, back test score and batch, and the random effects of individual pig. ^a^ Treatment Groups: M = castration + meloxicam, M + ID = castration + meloxicam + iron dextran, K = castration + ketoprofen, K + ID = castration + ketoprofen + iron dextran, C + ID = castration without analgesia + iron dextran, ID-C = iron dextran without castration, SH = sham handling without castration or injection. ^b^ Indicates a significant difference (*p* < 0.05) of treatment group from the referent category (C + ID). ^c^ 0 is equivalent to the average navigation time of the referent category (C + ID); error bars on the graph representative of a 95% confidence interval.

Overall navigation time was modeled controlling for time as a fixed-effect as well as accounting for a covariance structure of time between repeated measures. Other covariates that were controlled for included back test score, batch of observation, and baseline run time (the final recorded training run time). Overall navigation time did not differ between piglets treated with NSAID mixed with ID, and their respective NSAID given alone (model 1, Table 2 and Table 3). Overall navigation time (model 1, Figure 2, Table S3) was longer in C + ID piglets compared to all other treatment groups (*p* < 0.001). There were no significant differences between any other treatment groups for overall navigation time (*p* > 0.05) (model 1, see Table 2, Table 3, and Table S3). Analysis at the individual timepoints (model 2) showed that C + ID piglets had significantly longer navigation time than all other treatment groups at time = 0.25 h (*p* < 0.001). A significant difference between C + ID and all other groups was not found at any other timepoint. There were a few differences between individual treatment groups and C + ID (e.g., K + ID at 0.5 h, and 1 h), though no difference in non-castrated control groups (see Figures S1–S6). No other differences existed between the other treatment groups of interest for our research objectives at any specific timepoint (*p* > 0.05).

### 3.3. Cortisol (Model 3)

The C + ID treatment group had significantly higher levels of cortisol (nmol/L) 1 h post-castration compared to all other treatment groups (Table S6), when controlling for significant covariates of treatment day weight, castrator, and clustering at the litter level. There were no differences between M and M + ID groups (Table 4), nor were there differences between K and K + ID groups (Table 5). Groups that were castrated and given an NSAID in any form had similar cortisol levels compared to piglets that did not undergo castration, with the exception of M and M + ID piglets, where serum cortisol was higher (45.67 nmol/L, *p* = 0.027; and 45.45 nmol/L, *p* = 0.028; respectively) than the ID-C group. No cortisol differences were found between the M and M + ID groups and the other non-castrated SH group. There were no other differences between the treatment groups. One outlier with a very large standardized residual value was removed from analysis; it was noted that this pig was the only pig on trial euthanized due to health complications.

## 4. Discussion

The results of chute overall navigation time (model 1) showed no difference in average chute navigation times when piglets were given meloxicam or ketoprofen compounded with ID when compared to meloxicam or ketoprofen alone. These results also showed no difference in overall navigation time between piglets that were castrated and given either NSAID and the non-castrated control piglets (ID-C, SH). There was, however, a difference between piglets castrated without analgesia and all other treatment groups, in that piglets castrated without analgesia took significantly longer to navigate the chute. The lack of difference among the M, M + ID, K, and K + ID to ID-C and SH groups suggests that piglets given analgesia prior to castration, regardless of inclusion of ID in the formulation, had sufficient analgesia to navigate the chute in a comparable manner to piglets that were not castrated. In addition, the results provide evidence that the compounded formulations (M + ID and K + ID) retained pain control efficacy of the NSAIDs after being mixed with ID. The results are similar to Bilsborrow et al. [14], where piglets that were handled without castration, or castrated with meloxicam (4 mg/kg or 2 mg/kg) showed no difference in navigation time immediately post-castration. In addition, Bilsborrow et al. [14] found that both treatment groups given analgesia were significantly faster than piglets castrated without analgesia. One study evaluated ketoprofen and meloxicam using behavioral scoring as their outcome measure and found these NSAIDs to be ineffective to control castration pain [19]. However, these researchers made use of periodic video behavior scans and a piglet grimace scale (PGS) as the outcomes of interest [19]. The difference in results between our study and those of the study by Viscardi and Turner are likely due to methodological differences in how the studies were conducted [19]. In addition, recent research evaluating the PGS as an assessment tool noted no differences in average observer score between castrated and non-castrated pigs [20]. This suggests potential confounds with other stressors, which may indicate limited usefulness of the PGS for pain assessment in pigs.

Individual timepoint analysis of our data indicated a difference in C + ID from other treatment groups at the timepoint 0.25 h post-castration, and not at any other timepoint. This lack of difference in chute navigation at later timepoints may be due to a decreased pain sensation over time, decreased ability to measure pain with chute navigation at later timepoints, or a potential loss of NSAID effectiveness over time. Bilsborrow et al. [14] found similar results, documenting that piglets castrated with either a full or half-dose of meloxicam navigated the chute comparably to non-castrated piglets at 15 min post-castration. However, no differences were seen between any treatment groups past 15 min, measured four times between 30 min and 480 min post-castration [14]. 

Our statistical analysis differed from that of Bilsborrow et al. [14] as we analyzed all time points together in a single model, accounting for time as a fixed effect as well as for individual pig random effects. This allowed us to determine if there was a difference in treatment over the entire observation period independent of the effect of time and individual pig, which simple linear regression models at each timepoint do not account for. This highlights the importance of how we analyze pain outcome data, and the importance of controlling for other variables within our statistical model.

Another important observation in our study is that at later timepoints, there were no differences between non-analgesed castrates and non-castrated controls. This was also seen by Bilsborrow et al. [14]. This suggests that chute navigation is unable to quantify differences in pain at timepoints after an initial window post-castration, as we would expect non-castrated and castrated pigs to be significantly different.

It is unclear whether a lack of difference between treatment groups at individual timepoints past 15 min suggest a short duration of analgesia. Recent work by Nixon et al. [21] examined interstitial fluid (ISF) concentrations of both meloxicam and ketoprofen in 6 day old piglets. They found that while plasma concentrations rapidly decreased for these NSAIDs, and ISF concentrations remained above the limit of quantitation up to 48 h post-dosing [21]. This suggests that these NSAIDs would be available at the target site of action during the entire 30 h observation period in our study, and thus piglets given NSAIDs would likely have some level of analgesia at all timepoints. This supports that a lack of difference between treatment groups at timepoints past 15 min are likely due to the reduced ability to measure pain using the chute at those timepoints.

The administration of pain control 1 h prior to castration is not generally practical on-farm. The labor required on a large-scale swine operation to inject all pigs at a specific time interval before castration may not be feasible, and it is more likely that on commercial farms, analgesic injection would occur at the same time as castration (as well as other processing procedures). The objective of this study was to identify differences between pigs given NSAIDs alone when compared to pigs given NSAIDs compounded with ID, and not to assess specific timing of injection effects on analgesia. In order to assess differences in analgesia that may be present, it was an important study design consideration that animals that received NSAIDs were given enough time for the NSAIDs to be absorbed systemically prior to castration. That said, observation of preliminary pharmacokinetic data ([22] and Reynolds et al. (2020 unpublished)) indicates that the times to maximum serum concentration (T_max_) of meloxicam and ketoprofen compounded with ID occur within 2 h and 10 min, respectively. While the meloxicam compounded formulation showed a later T_max_ compared to ketoprofen, exposure of the body to the NSAID indicated by the area under the curve (AUC) during the drug absorption phase (time = 0 h until T_max_) may have been enough to provide analgesia. The results of these pharmacokinetic studies support that the NSAID drugs used may be available to the piglet at the target site, and available to provide analgesia shortly after castration, even if the injections are given within a short time frame of the castration procedure. Previous work has also shown that piglets given pre-emptive meloxicam 30 min prior to castration benefitted from post-operative analgesia [5,6]. Additional work by Nixon et al. in 6-day old piglets evaluating both meloxicam and ketoprofen suggest that optimal dosing may be around 2 h prior to castration for these NSAIDs as this correlates to peak ISF, which more closely represents analgesia at the target site [21]. As it is uncertain when the optimal time to administer NSAIDs would be, it would be worth exploring the optimal timing of NSAID injection prior to castration.

The results of cortisol analysis (model 3) support those of the chute navigation time analysis. Specifically, piglets in all castration treatment groups that received analgesia (M, M + ID, K, K + ID), and in non-castrated treatment groups (ID-C, SH) had significantly lower cortisol levels than C + ID pigs, suggesting that they may have experienced a reduced stress response. In addition, no differences were found between piglets given a specific NSAID vs. that NSAID compounded with ID. This supports that the NSAIDs meloxicam and ketoprofen, when compounded with ID, do not differ in the level of analgesia provided compared to the NSAID given alone, and that both formulations provide significant benefit to piglets compared to castration without analgesia. As noted previously, it is important to interpret these findings in relation to the timing of NSAID injection. A study by Sutherland et al. on biomarkers after castration showed no difference in serum cortisol between flunixin meglumine treated piglets and untreated piglets [23], however, these piglets were treated immediately prior to castration. Another study evaluating cortisol differences in piglets castrated with and without meloxicam also noted no change in post-castration cortisol [24], however, these piglets were not given meloxicam until after castration, and cortisol was measured at 20 min post-castration, which is before the ideal sampling period [18]. In addition, the pre-castration blood sample was taken 20 min prior to castration and may have elevated the post-castration sample [18]. It may be that the differences in cortisol found in our study were due to the injecting of NSAIDs 1 h prior to castration. Reductions in cortisol levels in pigs castrated with analgesia compared to animals castrated without analgesia have been found in other studies, which provided analgesia some time before castration such as Tenbergen et al. [6] where meloxicam was provided 30 min before castration, and Gottardo et al. [9] where meloxicam or ketoprofen was injected 10 min before castration. The cortisol differences between M and M + ID piglets to the ID-C group do not appear to show associated differences in chute navigation, and it is possible that these treatment group differences were not associated with differing levels of analgesia. While cortisol measurement is useful in studies of pain and distress, it has been shown that other factors may elevate levels in the blood, and results should be interpreted with caution [25,26].

The mixing of K or M with ID did not reduce the level of analgesia the piglets received. This information is helpful when working toward solutions for post-procedural pain control, while considering labor inputs and animal handling. While NSAIDs act to control post-procedural pain, it is important to recognize this does not address the acute pain associated with spermatic cord transection. Additional solutions need to be studied and incorporated into barn protocols to address this concern such as varying modes of anesthesia.

## 5. Conclusions

When looking at pain control as assessed by chute navigation time and serum cortisol levels, meloxicam and ketoprofen provided equivalent analgesia alone and when mixed with iron dextran. It appears that mixing these NSAIDs with iron dextran does not reduce analgesic efficacy. The compounded formulations could provide an added benefit of decreased labor, materials, and animal handling as well as comparable pain relief to current recommended medications. As compounding of veterinary licensed NSAIDs with ID is a novel practice, further work to investigate tissue drug depletion is required to ensure human food safety, prior to this practice being fully recommended.

## Figures and Tables

**Table 1:** Descriptive statistics of piglets (*n* = 25 per group) on trial by treatment group.

Parameter (Units)	M ^a^	M + ID	K	K + ID	C + ID	ID-C	SH
Mean age at treatment (d) (min–max)	3.3 (2–4)	3.4 (3–4)	3.3 (3–5)	3.4 (3–4)	3.4 (3–5)	3.3 (3–4)	3.4 (3–4)
Mean birth weight (kg) (min–max)	1.64 (1.12–2.49)	1.65 (1.17–2.19)	1.58 (0.97–2.41)	1.61 (1.03–2.26)	1.65 (1.13–2.34)	1.65 (1.04–2.34)	1.75 (1.02–2.32)
Mean treatment day weight (kg) (min–max)	2.14 (1.28–3.08)	2.09 (1.48–2.84)	2.00 (1.10–3.06)	1.96 (1.34–3.04)	2.10 (1.22–3.11)	2.16 (1.22–3.26)	2.27 (1.14–3.10)
Mean sow parity (min–max)	3.8 (1–7)	4.0 (1–8)	3.7 (1–7)	3.5 (1–7)	3.6 (1–7)	3.6 (1–7)	4.0 (1–7)
% cross fostered ^b^	15.8	21.1	31.6	31.6	26.3	31.6	31.6

^a^ Treatment Group (M = meloxicam and castrated, M + ID = meloxicam + iron dextran and castrated, K = ketoprofen and castrated, K + ID = ketoprofen + iron dextran and castrated, C + ID = castration only + iron dextran, ID-C = iron dextran without castration, SH = sham handled only). ^b^ Calculated as (piglets cross fostered in treatment group)/(all piglets in treatment group).

**Table 2:** Overall navigation time (seconds) by treatment group using meloxicam and iron dextran (M + ID) treatment group as the referent category, accounting for additional fixed ^a^ and random ^b^ effects (model 1) ^c^.

Variable	Coefficient	SE ^d^	*p* > |z|	95% Confidence Interval
Treatment ^e^				
M + ID	Referent	---	---	---
M	0.11	2.62	0.967	−5.02–5.23
K	0.87	2.63	0.742	−4.28–6.01
K + ID	−2.10	2.57	0.415	−7.13–2.94
C + ID	8.02	2.59	0.002	2.94–13.10
ID-C	1.54	2.55	0.547	−3.45–6.53
SH	−0.23	2.61	0.929	−5.35–4.88

^a^ Fixed effects include chute run time post castration, baseline navigation time, batch, and back test score. ^b^ Random effects include Individual Pig (Variance Estimate 8.35) and Residual: AR1 (Variance Estimate 180.92). ^c^ Mixed effects linear regression, estimated with restricted maximum likelihood estimation; full model output available as Table S1. ^e^ Treatment Groups: M = castration + meloxicam, M + ID = castration + meloxicam + iron dextran, K = castration + ketoprofen, K + ID = castration + ketoprofen + iron dextran, C + ID = castration without analgesia + iron dextran, ID-C = iron dextran without castration, SH = sham handling without castration or injection. ^d^ Standard Error.

**Table 3:** Overall navigation time (seconds) by treatment group using ketoprofen and iron dextran (K + ID) treatment group as the referent category, accounting for additional fixed ^a^ and random ^b^ effects (model 1) ^c^.

Variable	Coefficient	SE ^d^	*p* > |z|	95% Confidence Interval
Treatment ^e^				
K + ID	Referent	---	---	---
M	2.20	2.60	0.396	−2.88–7.30
M + ID	2.10	2.57	0.415	−2.94–7.14
K	2.96	2.61	0.256	−2.15–8.08
C + ID	10.12	2.58	<0.001	5.06–15.18
ID-C	3.63	2.59	0.160	−1.44–8.70
SH	1.86	2.61	0.475	−3.25–6.98

^a^ Fixed effects include chute run time post castration, baseline navigation time, batch, and back test score. ^b^ Random effects include Individual Pig (Variance Estimate 8.35) and Residual: AR1 (Variance Estimate 180.92). ^c^ Mixed effects linear regression, estimated with restricted maximum likelihood estimation; full model output available as Table S2. ^e^ Treatment Groups: M = castration + meloxicam, M + ID = castration + meloxicam + iron dextran, K = castration + ketoprofen, K + ID = castration + ketoprofen + iron dextran, C + ID = castration without analgesia + iron dextran, ID-C = iron dextran without castration, SH = sham handling without castration or injection. ^d^ Standard Error.

**Table 4:** Serum cortisol (nmol/L) 1-h post-castration by treatment group using meloxicam and iron dextran (M + ID) treatment group as the referent category, accounting for additional fixed ^a^ and random ^b^ effects (model 3) ^c^.

Variable	Coefficient	SE ^d^	*p* > |z|	95% Confidence Interval
Treatment ^e^				
M + ID	Referent	---	---	---
M	0.27	20.69	0.989	−40.29–40.83
K	−27.24	20.72	0.189	−67.85–13.37
K + ID	−23.08	20.76	0.266	−63.77–17.60
C + ID	77.82	20.92	<0.001	36.81–118.83
ID-C	−45.40	20.71	0.028	−85.98–−4.81
SH	−28.02	20.84	0.179	−68.86–12.83

^a^ Fixed effects include treatment day weight (per 100 g), and individual castrator. ^b^ Random effects include litter Individual Pig (variance estimate 1544.32, variance partition coefficient = 0.224). ^c^ Mixed effects linear regression; full model output available as Table S4. ^e^ Treatment Groups: M = castration + meloxicam, M + ID = castration + meloxicam + iron dextran, K = castration + ketoprofen, K + ID = castration + ketoprofen + iron dextran, C + ID = castration without analgesia + iron dextran, ID-C = iron dextran without castration, SH = sham handling without castration or injection. ^d^ Standard Error.

**Table 5:** Serum cortisol (nmol/L) 1-h post-castration by treatment group using ketoprofen and iron dextran (K + ID) treatment group as the referent category, accounting for additional fixed ^a^ and random ^b^ effects (model 3) ^c^.

Variable	Coefficient	SE ^d^	*p* > |z|	95% Confidence Interval
Treatment ^e^				
K + ID	Referent	---	---	---
M	23.36	20.83	0.262	−17.47–64.18
M + ID	23.08	20.76	0.266	−17.60–63.77
K	−4.16	20.69	0.841	−44.71–36.39
C + ID	100.90	21.04	<0.001	59.66–142.15
ID-C	−22.31	20.87	0.285	−63.22–18.60
SH	−4.93	21.13	0.815	−46.35–36.49

^a^ Fixed effects include treatment day weight (per 100 g), and individual castrator. ^b^ Random effects include litter (variance estimate 1544.32, variance partition coefficient = 0.224). ^c^ Mixed effects linear regression; full model output available as Table S5. ^e^ Treatment Groups: M = castration + meloxicam, M + ID = castration + meloxicam + iron dextran, K = castration + ketoprofen, K + ID = castration + ketoprofen + iron dextran, C + ID = castration without analgesia + iron dextran, ID-C = iron dextran without castration, SH = sham handling without castration or injection. ^d^ Standard Error.

## Supplementary Materials

The following are available online at https://www.mdpi.com/2076-2615/10/7/1237/s1, Table S1: Final full model (model 1) of overall navigation time (seconds) by treatment group using meloxicam and iron dextran (M + ID) treatment group as the referent category, Table S2: Final full model (model 1) of overall navigation time (seconds) by treatment group using ketoprofen and iron dextran (K + ID) treatment group as the referent category, Table S3: Final full model (model 1) of overall navigation time (seconds) by treatment group using castration without analgesia and iron dextran (C + ID) treatment group as the referent category, Table S4: Final full model (model 3) of cortisol (nmol/L) 1-h post-castration by treatment group using meloxicam and iron dextran (M + ID) treatment group as the referent category, Table S5: Final full model (model 3) of cortisol (nmol/L) 1-h post-castration by treatment group using ketoprofen and iron dextran (K + ID) treatment group as the referent category, Table S6: Final full model (model 3) of cortisol (nmol/L) 1-h post-castration by treatment group using castration without analgesia and iron dextran (C + ID) treatment group as the referent category, Figure S1. Comparison of navigation time (sec) at 15 min post-castration between treatment groups using simple linear regression, controlling for fixed effects of navigation timepoint, baseline navigation time, back test score and batch, Figure S2. Comparison of navigation time (sec) at 30 min post-castration between treatment groups using simple linear regression, controlling for fixed effects of navigation timepoint, baseline navigation time, back test score and batch, Figure S3. Comparison of navigation time (sec) at 1 h post-castration between treatment groups using simple linear regression, controlling for fixed effects of navigation timepoint, baseline navigation time, back test score and batch, Figure S4. Comparison of navigation time (sec) at 4 h post-castration between treatment groups using simple linear regression, controlling for fixed effects of navigation timepoint, baseline navigation time, back test score and batch, Figure S5. Comparison of navigation time (sec) at 24 h post-castration between treatment groups using simple linear regression, controlling for fixed effects of navigation timepoint, baseline navigation time, back test score and batch, Figure S6. Comparison of navigation time (sec) at 30 h post-castration between treatment groups using simple linear regression, controlling for fixed effects of navigation timepoint, baseline navigation time, back test score and batch.

## References

[1] (2014). National Farm Animal Care Council. http://www.nfacc.com/pdfs/codes/pig_code_of_practice.pdf.

[2] Hansson M., Lundeheim N., Nyman G., Johansson G. (2011). Effect of local anesthesia and/or analgesia on pain responses induced by piglet castration. Acta Vet. Scand..

[3] Hay M., Vulin A., Génin S., Sales P., Prunier A. (2003). Assessment of pain induced by castration in piglets: Behavioural and physiological responses over the subsequent 5 days. Appl. Anim. Behav. Sci..

[4] Kluivers-Poodt M., Zonderland J.J., Verbraak J., Lambooij E., Hellebrekers L.J. (2013). Pain behaviour after castration of piglets; effect of pain relief with lidocaine and/or meloxicam. Animal.

[5] Keita A., Pagot E., Prunier A., Guidarini C. (2010). Pre-emptive meloxicam for postoperative analgesia in piglets undergoing surgical castration. Vet. Anaesth. Analg..

[6] Tenbergen R., Friendship R., Cassar G., Amezcua M.R., Haley D. (2014). Investigation of the use of meloxicam for reducing pain associated with castration and tail docking and improving performance in piglets. J. Swine Health Prod..

[7] Numberger J., Ritzmann M., Übel N., Eddicks M., Reese S., Zöls S. (2016). Ear tagging in piglets: The cortisol response with and without analgesia in comparison with castration and tail docking. Animal.

[8] Courboulay V., Hemonic A., Gadonna M., Prunier A. (2010). Castration avec anesthesie locale ou traitement anti-inflammatoire: Quel impact sur la douleur des porcelets et quelles consequences sur le travail en elevage?. Journ. Rech. Porc..

[9] Gottardo F., Scollo A., Contiero B., Ravagnani A., Tavella G., Bernardini D., De Benedictis G.M., Edwards S.A. (2016). Pain alleviation during castration of piglets: A comparative study of different farm options. J. Anim. Sci..

[10] Perri A.M., Friendship R.M., Harding J.C.S., O’Sullivan T.L. (2016). An investigation of iron deficiency and anemia in piglets and the effect of iron status at weaning on post-weaning performance. J. Swine Health Prod..

[11] Health Canada Policy on Extra-Label Drug Use (ELDU) in Food Producing Animals. https://www.canada.ca/content/dam/hc-sc/migration/hc-sc/dhp-mps/alt_formats/hpfb-dgpsa/pdf/vet/pol_eldu-umdde-eng.pdf.

[12] Cribb A.E., Peryou M. (2006). Small Animal Toxicology.

[13] Barz A., Ritzmann M., Breitinger I., Langhoff R., Zöls S., Palzer A., Heinritzi K. (2010). Examination of different options for combined administration of an NSAID (meloxicam) and iron for piglets being castrated. Tierärztliche Prax. Großtiere.

[14] Bilsborrow K., Seddon Y.M., Brown J., Waldner C., Stookey J.M. (2016). An investigation of a novel behavioural test to assess pain in piglets following castration. Can. J. Anim. Sci..

[15] Davis K., Seddon Y., Creutzinger K., Bouvier M., Brown J. (2017). An investigation into the use of sucrose to reduce castration pain in piglets. Can. J. Anim. Sci..

[16] Hessing M.J.C., Hagelsø A.M., van Beek J.A.M., Wiepkema R.P., Schouten W.G.P., Krukow R. (1993). Individual behavioural characteristics in pigs. Appl. Anim. Behav. Sci..

[17] Huhn R.G., Osweiller G.D., Switzer W.P. (1969). Application of the orbital sinus bleeding technique to swine. Lab. Anim. Care.

[18] Carroll J.A., Berg E.L., Strauch T.A., Roberts M.P., Kattesh H.G. (2006). Hormonal profiles, behavioral responses, and short-term growth performance after castration of pigs at three, six, nine, or twelve days of age. J. Anim. Sci..

[19] Viscardi A.V., Turner P.V. (2018). Use of meloxicam or ketoprofen for piglet pain control following surgical castration. Front. Vet. Sci..

[20] Di Giminiani P., Brierley V.L.M.M., Scollo A., Gottardo F., Malcolm E.M., Edwards S.A., Leach M.C. (2016). The assessment of facial expressions in piglets undergoing tail docking and castration: Toward the development of the piglet grimace scale. Front. Vet. Sci..

[21] Nixon E., Almond G.W., Baynes R.E., Messenger K.M. (2020). Comparative plasma and interstitial fluid pharmacokinetics of meloxicam, flunixin and ketoprofen in neonatal pigs. Front. Vet. Sci..

[22] Ramkissoon S., O’Sullivan T., DeLay J., Enouri S., Friendship R., Johnson R. Mixing (compounding) iron dextran with NSAIDs for use in piglets. Proceedings of the 34th Annual Centralia Swine Research Update.

[23] Sutherland M.A., Davis B.L., Brooks T.A., Coetzee J.F. (2012). The physiological and behavioral response of pigs castrated with and without anesthesia or analgesia. J. Anim. Sci..

[24] Bonastre C., Mitjana O., Tejedor M.T., Calavia M., Yuste A.G., Úbeda J.L., Falceto M.V. (2016). Acute physiological responses to castration-related pain in piglets: The effect of two local anesthetics with or without meloxicam. Animal.

[25] Mormède P., Andanson S., Aupérin B., Beerda B., Guémené D., Malmkvist J., Manteca X., Manteuffel G., Prunet P., Van Reenen C.G. (2007). Exploration of the hypothalamic-pituitary-adrenal function as a tool to evaluate animal welfare. Physiol. Behav..

[26] Désautés C., Bidanel J.P., Mormède P. (1997). Genetic study of behavioural and pituitary-adrenocortical reactivity in response to an environmental challenge in pigs. Physiol. Behav..

