# Supplementary material for: Assessing Pain Control Efficacy of Meloxicam and Ketoprofen When Compounded with Iron Dextran in Nursing Piglets Using A Navigation Chute
Source: Animals (Basel). 2020 Jul 21;10(7):1237. doi: 10.3390/ani10071237 (PMC7401524; doi:10.3390/ani10071237)
Supplement: Supplementary file 1 [file animals-10-01237-s001.pdf]

Manuscript 850100 – Supplementary Materials

Table S1. Final full model* (model 1) of overall navigation time (sec) by treatment group using meloxicam and iron dextran (M+ID) treatment group as the referent category.

Variable	Coefficient	SE[a]	z	$p > \|z\|$	95% Confidence Interval
			FIXED EFFECTS		
Treatment[b]					
M+ID	*Referent*	---	---	---	---
M	0.11	2.62	0.04	0.967	-5.02 - 5.23
K	0.87	2.63	0.33	0.742	-4.28 - 6.01
K+ID	-2.10	2.57	-0.82	0.415	-7.13 - 2.94
C+ID	8.02	2.59	3.09	0.002	2.94 - 13.10
ID-C	1.54	2.55	0.60	0.547	-3.45 - 6.53
SH	-0.23	2.61	-0.09	0.929	-5.35 - 4.88
Chute Run Time Post-Castration (h)	-0.26	0.04	-7.03	<0.001	-0.33 - -0.19
Baseline Navigation Time (sec)	0.26	0.06	4.14	<0.001	0.14 - 0.38
Batch					
Mid-June	*Referent*	---	---	---	---
Mid-July	-5.52	2.49	-2.22	0.026	-10.40 - -0.65
Early August	-6.65	2.49	-2.67	0.008	-11.53 - -1.78
Late August	-7.15	2.49	-2.87	0.004	-12.04 - -2.26
Late September	-5.58	2.41	-2.32	0.020	-10.30 - -0.87
Back Test Score					
0	*Referent*	---	---	---	---
1	-2.81	1.67	-1.68	0.093	-6.08 - 0.47
2	-4.16	1.82	-2.29	0.022	-7.73 - -0.60
(Constant)	20.70	2.95	7.01	<0.001	14.91 - 26.49
			RANDOM EFFECTS		
**Variable**	**Variance Estimate**	**SE[a]**			**95% Confidence Interval**
Individual Pig	8.35	20.65			0.07 - 1063.93
Residual: AR1	180.92	21.54			143.262 - 228.48

*Mixed effects linear regression; estimated with restricted maximum likelihood estimation. [a]Standard Error [b]Treatment Groups: M = castration + meloxicam, M+ID = castration + meloxicam + iron dextran, K = castration + ketoprofen, K+ID = castration + ketoprofen + iron dextran, C+ID = castration without analgesia + iron dextran, ID-C = iron dextran without castration, SH = sham handling without castration or injection.

Table S2. Final full model* (model 1) of overall navigation time (sec) by treatment group using ketoprofen and iron dextran (K+ID) treatment group as the referent category.

Variable	Coefficient	SE[a]	z	*p* > |z|	95% Confidence Interval
			FIXED EFFECTS		
Treatment[b]					
K+ID	*Referent*	---	---	---	---
M	2.20	2.60	0.85	0.396	-2.88 - 7.30
M+ID	2.10	2.57	0.82	0.415	-2.94 - 7.14
K	2.96	2.61	1.13	0.256	-2.15 - 8.08
C+ID	10.12	2.58	3.92	<0.001	5.06 - 15.18
ID-C	3.63	2.59	1.40	0.160	-1.44 - 8.70
SH	1.86	2.61	0.71	0.475	-3.25 - 6.98
Chute Run Time Post-Castration (h)	-0.26	0.04	-7.03	<0.001	-0.33 - -0.19
Baseline Navigation Time (sec)	0.26	0.06	4.14	<0.001	0.14 - 0.38
Batch					
Mid-June	*Referent*	---	---	---	---
Mid-July	-5.52	2.49	-2.22	0.026	-10.40 - -0.65
Early August	-6.65	2.49	-2.67	0.008	-11.53 - -1.78
Late August	-7.15	2.49	-2.87	0.004	-12.04 - -2.26
Late September	-5.58	2.41	-2.32	0.020	-10.30 - -0.87
Back Test Score					
0	*Referent*	---	---	---	---
1	-2.81	1.67	-1.68	0.093	-6.08 - 0.47
2	-4.16	1.82	-2.29	0.022	-7.73 - -0.60
(Constant)	18.61	3.12	5.95	<0.001	12.48 - 24.73
			RANDOM EFFECTS		
**Variable**	**Variance Estimate**	**SE[a]**			**95% Confidence Interval**
Individual Pig	8.35	20.65			0.07 - 1063.93
Residual: AR1	180.92	21.54			143.262 - 228.48

*Mixed effects linear regression; estimated with restricted maximum likelihood estimation. [a]Standard Error [b]Treatment Groups: M = castration + meloxicam, M+ID = castration + meloxicam + iron dextran, K = castration + ketoprofen, K+ID = castration + ketoprofen + iron dextran, C+ID = castration without analgesia + iron dextran, ID-C = iron dextran without castration, SH = sham handling without castration or injection.

Table S3. Final full model* (model 1) of overall navigation time (sec) by treatment group using castration without analgesia and iron dextran (C+ID) treatment group as the referent category.

Variable	Coefficient	SE[a]	z	$p > \|z\|$	95% Confidence Interval
			FIXED EFFECTS		
Treatment[b]					
C+ID	*Referent*	---	---	---	---
M	-7.91	2.61	-3.03	0.002	-13.04 - -2.79
M+ID	-8.02	2.59	-3.09	0.002	-13.10 - -2.94
K	-7.15	2.65	-2.70	0.007	-12.35 - -1.97
K+ID	-10.12	2.58	-3.92	0.000	-15.18 - -5.06
ID-C	-6.48	2.60	-2.49	0.013	-11.58 - -1.39
SH	-8.26	2.63	-3.14	0.002	-13.42 - -3.10
Chute Run Time Post-Castration (hrs)	-0.26	0.04	-7.03	<0.001	-0.33 - -0.19
Baseline Navigation Time (sec)	0.26	0.06	4.14	<0.001	0.14 - 0.38
Batch					
Mid-June	*Referent*	---	---	---	---
Mid-July	-5.52	2.49	-2.22	0.026	-10.40 - -0.65
Early August	-6.65	2.49	-2.67	0.008	-11.53 - -1.78
Late August	-7.15	2.49	-2.87	0.004	-12.04 - -2.26
Late September	-5.58	2.41	-2.32	0.020	-10.30 - -0.87
Back Test Score					
0	*Referent*	---	---	---	---
1	-2.81	1.67	-1.68	0.093	-6.08 - 0.47
2	-4.16	1.82	-2.29	0.022	-7.73 - -0.60
(Constant)	28.72	3.06	9.39	<0.001	22.73 - 34.72
			RANDOM EFFECTS		
**Variable**	**Variance Estimate**	**SE[a]**			**95% Confidence Interval**
Individual Pig	8.35	20.65			0.07 - 1063.93
Residual: AR1	180.92	21.54			143.262 - 228.48

*Mixed effects linear regression; estimated with restricted maximum likelihood estimation. [a]Standard Error. [b]Treatment Groups: M = castration + meloxicam, M+ID = castration + meloxicam + iron dextran, K = castration + ketoprofen, K+ID = castration + ketoprofen + iron dextran, C+ID = castration without analgesia + iron dextran, ID-C = iron dextran without castration, SH = sham handling without castration or injection.

Table S4. Final full model* (model 3) of cortisol (nmol/L) 1-h post-castration by treatment group using meloxicam and iron dextran (M+ID) treatment group as the referent category.

Variable	Coefficient	SE[a]	z	$p > \|z\|$	95% Confidence Interval
			FIXED EFFECTS		
Treatment[b]					
M+ID	*Referent*	---	---	---	---
M	0.27	20.69	0.01	0.989	-40.29 - 40.83
K	-27.24	20.72	-1.31	0.189	-67.85 - 13.37
K+ID	-23.08	20.76	-1.11	0.266	-63.77 - 17.60
C+ID	77.82	20.92	3.72	<0.001	36.81 - 118.83
ID-C	-45.40	20.71	-2.19	0.028	-85.98 - -4.81
SH	-28.02	20.84	-1.34	0.179	-68.86 - 12.83
Treatment Day Weight (per 100g)	-5.59	1.40	-4.00	<0.001	-8.33 - -2.85
Castrator					
A	*Referent*	---	---	---	---
B	-124.57	36.36	-3.43	0.001	-195.83 - -53.31
C	-116.05	38.61	-3.01	0.003	-191.72 - -40.39
D	-163.30	48.04	-3.40	0.001	-257.47 - -69.14
(Constant)	433.39	46.24	9.37	<0.001	342.77 - 524.01
			RANDOM EFFECTS		
**Variable**	**Variance Estimate**	**SE**[a]			**95% Confidence Interval**
Litter	1544.32[c]	678.76			652.55 - 3654.74

*Linear Mixed model, accounting for clustering at the litter level, and controlling for covariates of treatment day weight (dg), and individual castrator effect [a]Standard Error [b]Treatment Groups: M = castration + meloxicam, M+ID = castration + meloxicam + iron dextran, K = castration + ketoprofen, K+ID = castration + ketoprofen + iron dextran, C+ID = castration without analgesia + iron dextran, ID-C = iron dextran without castration, SH = sham handling without castration or injection. [c]Variance Partition Coefficient of Litter = 0.224.

Table S5. Final full model* (model 3) of cortisol (nmol/L) 1-hr post-castration by treatment group using ketoprofen and iron dextran (K+ID) treatment group as the referent category.

Variable	Coefficient	SE[a]	z	$p > \lvert z \rvert$	95% Confidence Interval
			FIXED EFFECTS		
Treatment[b]					
K+ID	*Referent*	---	---	---	---
M	23.36	20.83	1.12	0.262	-17.47 - 64.18
M+ID	23.08	20.76	1.11	0.266	-17.60 - 63.77
K	-4.16	20.69	-0.20	0.841	-44.71 - 36.39
C+ID	100.90	21.04	4.80	<0.001	59.66 - 142.15
ID-C	-22.31	20.87	-1.07	0.285	-63.22 - 18.60
SH	-4.93	21.13	-0.23	0.815	-46.35 - 36.49
Treatment Day Weight (per 100g)	-5.59	1.40	-4.00	<0.001	-8.33 - -2.85
Castrator					
A	*Referent*	---	---	---	---
B	-124.57	36.36	-3.43	0.001	-195.83 - -53.31
C	-116.05	38.61	-3.01	0.003	-191.72 - -40.39
D	-163.30	48.04	-3.40	0.001	-257.47 - -69.14
(Constant)	433.39	46.24	9.37	<0.001	342.77 - 524.01
			RANDOM EFFECTS		
**Variable**	**Variance Estimate**	**SE[a]**			**95% Confidence Interval**
Litter	1544.32[c]	678.76			652.55 - 3654.74

*Linear Mixed model, accounting for clustering at the litter level, and controlling for covariates of treatment day weight (dg), and individual castrator effect [a]Standard Error [b]Treatment Groups: M = castration + meloxicam, M+ID = castration + meloxicam + iron dextran, K = castration + ketoprofen, K+ID = castration + ketoprofen + iron dextran, C+ID = castration without analgesia + iron dextran, ID-C = iron dextran without castration, SH = sham handling without castration or injection. [c]Variance Partition Coefficient of Litter = 0.224.

Table S6. Final full model* (model 3) of cortisol (nmol/L) 1-h post-castration by treatment group using castration without analgesia and iron dextran (C+ID) treatment group as the referent category.

Variable	Coefficient	SE[a]	z	$p > \lvert z \rvert$	95% Confidence Interval
		FIXED EFFECTS			
***Treatment*[b]**					
C+ID	*Referent*	---	---	---	---
M	-77.55	20.92	-3.71	<0.001	-118.55 - -36.54
M+ID	-77.82	20.92	-3.72	<0.001	-118.83 - -36.81
K	-105.06	20.99	-5.01	<0.001	-146.20 - -63.92
K+ID	-100.90	21.04	-4.80	<0.001	-142.15 - -59.66
ID-C	-123.22	20.93	-5.89	<0.001	-164.23 - -82.20
SH	-105.84	21.02	-5.03	<0.001	-147.04 - -64.64
Treatment Day Weight (per 100g)	-5.59	1.40	-4.00	<0.001	-8.33 - -2.85
Castrator					
A	*Referent*	---	---	---	---
B	-124.57	36.36	-3.43	0.001	-195.83 - -53.31
C	-116.05	38.61	-3.01	0.003	-191.72 - -40.39
D	-163.30	48.04	-3.40	0.001	-257.47 - -69.14
(Constant)	433.39	46.24	9.37	<0.001	342.77 - 524.01
		RANDOM EFFECTS			
**Variable**	**Variance Estimate**	**SE[a]**			**95% Confidence Interval**
Litter	1544.32[c]	678.76			652.55 - 3654.74

*Linear Mixed model, accounting for clustering at the litter level, and controlling for covariates of treatment day weight (dg), and individual castrator effect; [a]Standard Error; [b]Treatment Groups: M = castration + meloxicam, M+ID = castration + meloxicam + iron dextran, K = castration + ketoprofen, K+ID = castration + ketoprofen + iron dextran, C+ID = castration without analgesia + iron dextran, ID-C = iron dextran without castration, SH = sham handling without castration or injection; [c]Variance Partition Coefficient of Litter = 0.224.

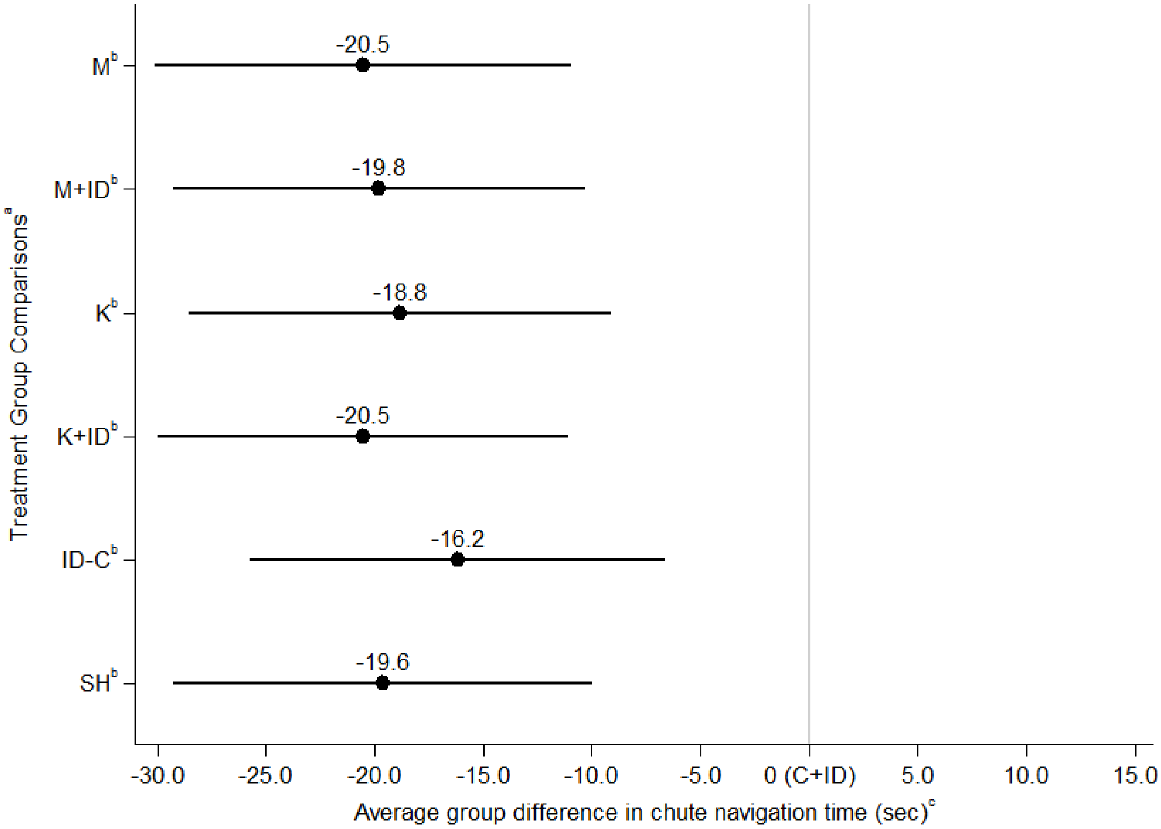

Figure S1. Comparison of navigation time (sec) at 15 min post-castration between treatment groups using simple linear regression, controlling for fixed effects of navigation timepoint, baseline navigation time, back test score and batch. [a]Treatment Groups: M = castration + meloxicam, M+ID = castration + meloxicam + iron dextran, K = castration + ketoprofen, K+ID = castration + ketoprofen + iron dextran, C+ID = castration without analgesia + iron dextran, ID-C = iron dextran without castration, SH = sham handling without castration or injection. [b]Indicates a significant difference ($P<0.05$) of treatment group from the referent category (C+ID). [c]0 is equivalent to the average navigation time of the referent category (C+ID); error bars on the graph representative of a 95% confidence interval.

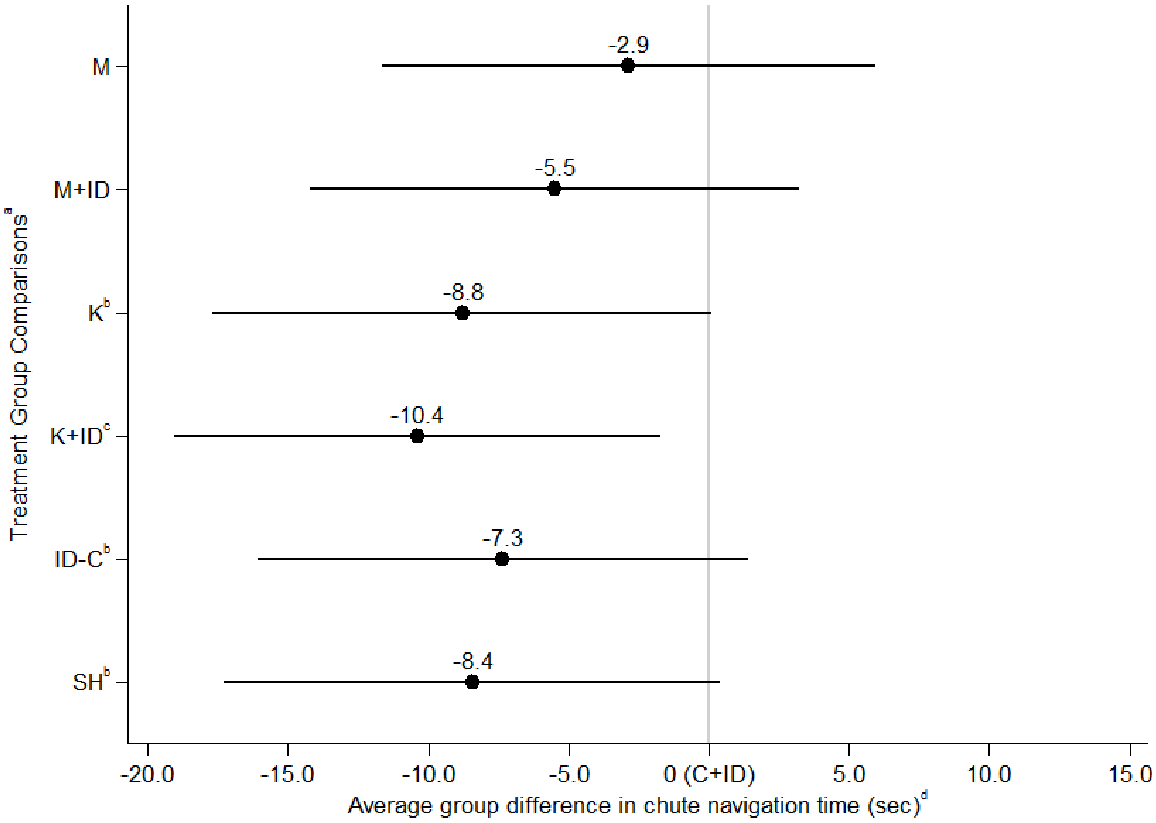

Figure S2. Comparison of navigation time (sec) at 30 min post-castration between treatment groups using simple linear regression, controlling for fixed effects of navigation timepoint, baseline navigation time, back test score and batch. [a]Treatment Groups: M = castration + meloxicam, M+ID = castration + meloxicam + iron dextran, K = castration + ketoprofen, K+ID = castration + ketoprofen + iron dextran, C+ID = castration without analgesia + iron dextran, ID-C = iron dextran without castration, SH = sham handling without castration or injection. [b]Indicates a trend in difference (P=0.05-0.10) of treatment group from the referent category (C+ID). [c]Indicates a significant difference (P<0.05) of treatment group from the referent category (C+ID). [d]0 is equivalent to the average navigation time of the referent category (C+ID); error bars on the graph representative of a 95% confidence interval.

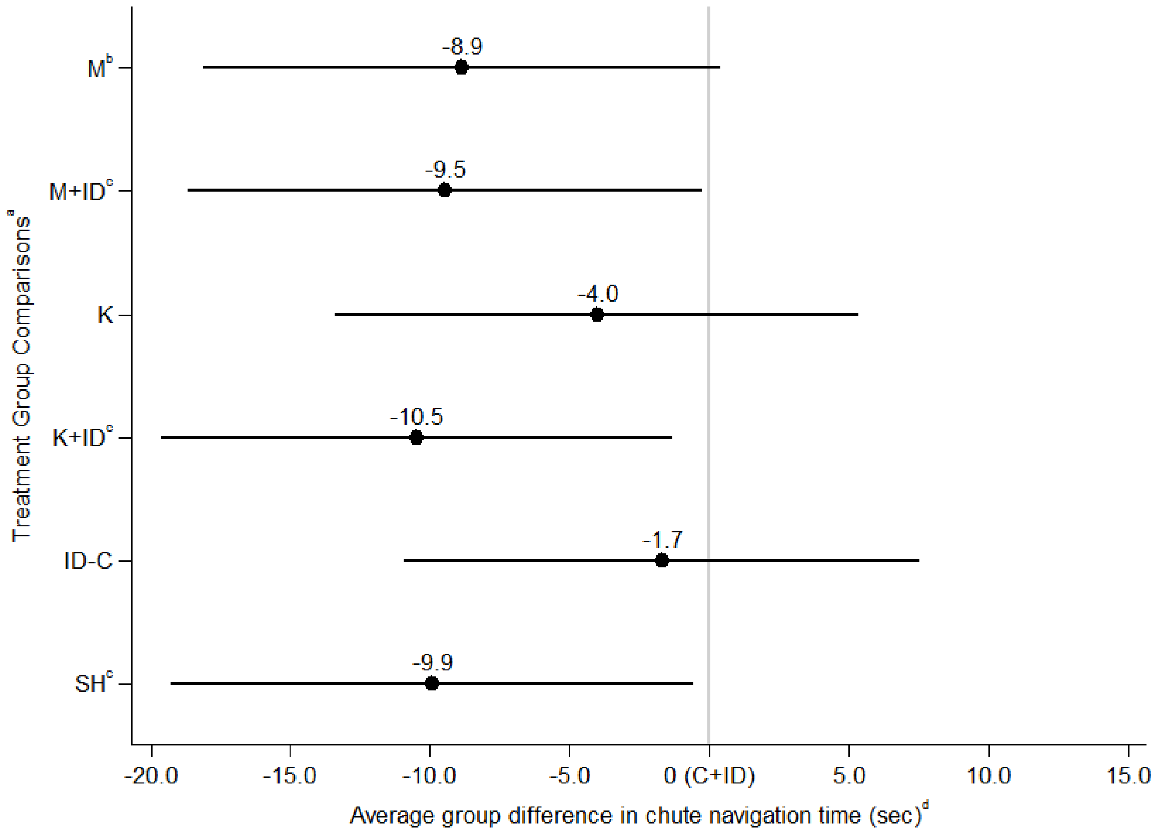

Figure S3. Comparison of navigation time (sec) at 1 h post-castration between treatment groups using simple linear regression, controlling for fixed effects of navigation timepoint, baseline navigation time, back test score and batch. [a]Treatment Groups: M = castration + meloxicam, M+ID = castration + meloxicam + iron dextran, K = castration + ketoprofen, K+ID = castration + ketoprofen + iron dextran, C+ID = castration without analgesia + iron dextran, ID-C = iron dextran without castration, SH = sham handling without castration or injection. [b]Indicates a trend in difference (P=0.05-0.10) of treatment group from the referent category (C+ID). [c]Indicates a significant difference (P<0.05) of treatment group from the referent category (C+ID). [d]0 is equivalent to the average navigation time of the referent category (C+ID); error bars on the graph representative of a 95% confidence interval.

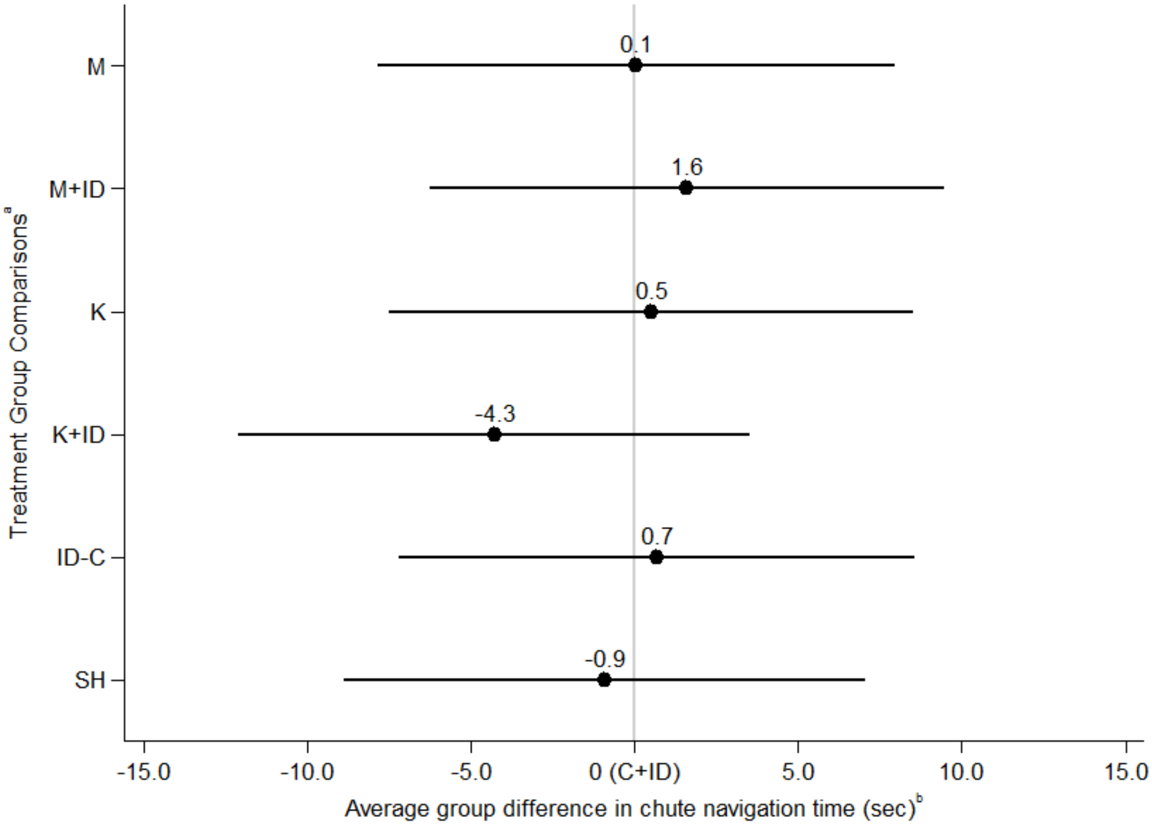

Figure S4. Comparison of navigation time (sec) at 4 h post-castration between treatment groups using simple linear regression, controlling for fixed effects of navigation timepoint, baseline navigation time, back test score and batch. [a]Treatment Groups: M = castration + meloxicam, M+ID = castration + meloxicam + iron dextran, K = castration + ketoprofen, K+ID = castration + ketoprofen + iron dextran, C+ID = castration without analgesia + iron dextran, ID-C = iron dextran without castration, SH = sham handling without castration or injection. [b]0 is equivalent to the average navigation time of the referent category (C+ID); error bars on the graph representative of a 95% confidence interval.

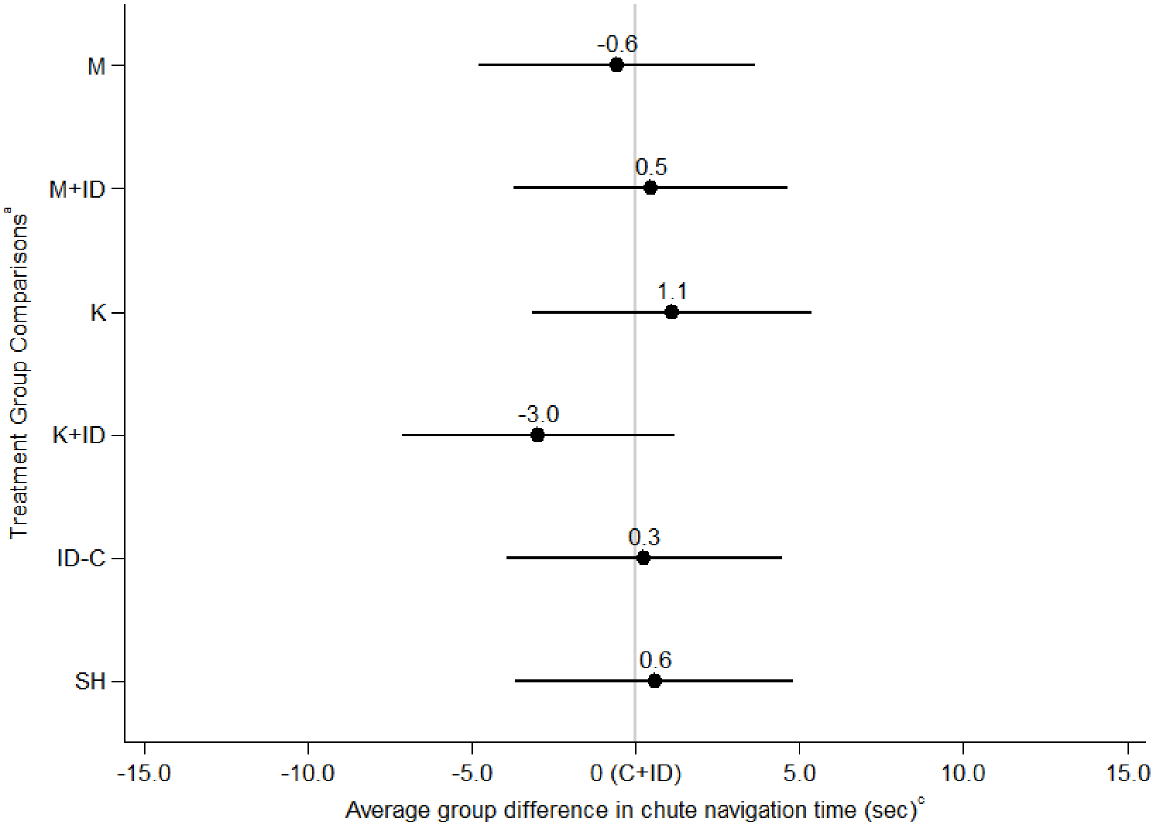

Figure S5. Comparison of navigation time (sec) at 24 h post-castration between treatment groups using simple linear regression, controlling for fixed effects of navigation timepoint, baseline navigation time, back test score and batch. [a]Treatment Groups: M = castration + meloxicam, M+ID = castration + meloxicam + iron dextran, K = castration + ketoprofen, K+ID = castration + ketoprofen + iron dextran, C+ID = castration without analgesia + iron dextran, ID-C = iron dextran without castration, SH = sham handling without castration or injection. [b]0 is equivalent to the average navigation time of the referent category (C+ID); error bars on the graph representative of a 95% confidence interval.

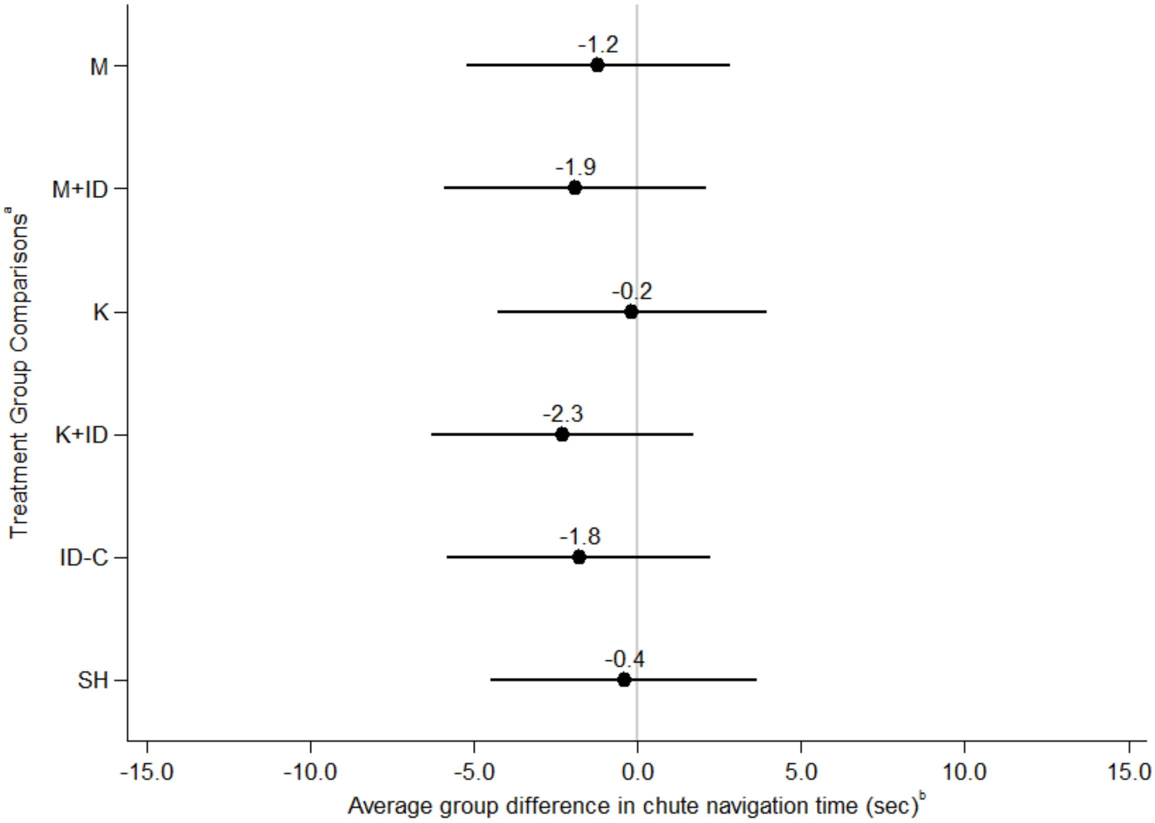

Figure S6. Comparison of navigation time (sec) at 30 h post-castration between treatment groups using simple linear regression, controlling for fixed effects of navigation timepoint, baseline navigation time, back test score and batch. [a]Treatment Groups: M = castration + meloxicam, M+ID = castration + meloxicam + iron dextran, K = castration + ketoprofen, K+ID = castration + ketoprofen + iron dextran, C+ID = castration without analgesia + iron dextran, ID-C = iron dextran without castration, SH = sham handling without castration or injection. [b]0 is equivalent to the average navigation time of the referent category (C+ID); error bars on the graph representative of a 95% confidence interval.
